# Professional Honesty and Decision of Character in the Practice of Dentistry

**Published:** 1902-02

**Authors:** N. G. Carroll

**Affiliations:** North Carolina


### PROFESSIONAL HONESTY AND DECISION OF CHARACTER IN THE PRACTICE OF DENTISTRY.[1]

BY DR. N. G. CARROLL, NORTH CAROLINA.

In dental practice there are many small attentions, apparently trifling in themselves, that impress your patients with your care and consideration for their comfort, and, as far as the eye can influence, they are assured of cleanliness and neatness of operating. I refer

[1] Read before the North Carolina State Dental Association, June, 1901.

principally to the essentials of proper personal appearance, clean office and instruments, which are often accepted as indicative of the character of a practitioner. But I wish to go back of this and throw out a few hints as to "Professional Honesty and Decision of Character in the Practice of Dentistry." As you follow me along, bear in mind "honor and glory" are not nourishing, but to the true professional man dearer than wealth.

As our guide, let us choose the motto, "The best Capital is Character."

I suggest this guide because it is an unselfish one. It will tell you every dentist has two reputations,—one among his professional brethren and one with the public. Character is one of the greatest motive powers in the world. In its noblest embodiments, it exemplifies human nature in its highest forms, for it exhibits man at his best. Men of honor and character are the conscience of the society or profession to which they belong. Martin Luther said, "The prosperity of a country depends, not on the abundance of its revenues, nor on the strength of its fortifications, nor on the beauty of its public buildings; but it consists in the number of its cultivated citizens, in its men of education, enlightenment, honor, and character; here are to be found its true interests, its chief strength, its real power." The practice of dentistry is one of the most noble callings of this advanced age, and yet I know of no other calling or vocation of which the outside world or people in general have so limited a knowledge as to its detail and minute practice and operations. It may be safely said that every man who occupies a professional position declares to the world that he possesses as much knowledge and skill as may be necessary for the accomplishment of any operation within the sphere of his calling. This truth applies as well in the law as in common sense. In a trade the article is sought, it can be examined, and its value estimated, while in a profession the public seeks the individual, having confidence in his honesty and ability. Therefore, when a patient seeks or asks for your services, it is a declaration of confidence and trust. Hence, a man who follows the profession should be made of fibre tough enough to stand by principle and maintain the professional honor and decision of character that so nobly, justly, and truly belong to this profession in which providence has placed him. This doing of one's duty embodies the highest ideal of life and character. He will then have served his gifts and profession—*not abused them.*

But while our ablest and most energetic members of the profession, our geniuses full of enthusiasm for the progress and advancement of dentistry, are continually advancing and are striving to lead us to a broader sphere of usefulness and to a higher scientific knowledge, and we as a professional body with sincere motives and earnest desire to be useful to our fellow-man and honor to our profession, striving on, falling short in many respects, though possibly doing as well as should be expected; what about the advertising strugglers, quacks and scabs who shirk the principles of professional honesty and with dormant sense of right and wrong, with stifled conscience and no regard for ethics, but are eager to rush in and push themselves into an injurious prominence. The question of advertising is one that must be decided on entering practice, and often with the young graduate the questions of immediate gain, or future gain, with professional honor and respectability, are trying ones.

The man who is seeking the road to wealth is misdirected when he chooses the dental profession, though the average dentist lives well and comfortably, spends money freely, but dies without attaining great wealth. I hope I am not cynical on the subject of advertising, and I see no objection to a man doing so in a professional manner. To have been heard of, if only once, is a great advantage with the new practitioner, and the young man just entering the profession realizes this fact, and while it is his desire to conduct himself in a professional manner and live up to the code of ethics, he may feel some necessity to bring his name before the public. Should the young man desire to simply advertise his name, calling, and address in some reputable daily or weekly paper which is not used by the quack, I can see no special objection. Such advertisting cannot be expected to yield immediate or direct results; the effect or desire is simply that the community or public become familiar with his name, calling, and address. But be sure and stop with the name and address, do not so much as say "examinations free." While I say there can be no serious objections to this form of advertising, the reputable dentist, feeling there must be a dividing line between him and the incompetent, refuses to advertise at all. The advancement of dentistry has not come through the advertising offices; they are simply parasites, who live on the work of others, pulling them down by deceiving the public; they contribute nothing to the upbuilding or advancement of the profession or the investigation of new problems. The man who advertises a "dental parlor" and superiority over all his professional brethren is to be avoided; likewise, the man who seeks the medium of advertising cheap work through the daily papers and theatre programmes, for if his services were worth more, he could command a higher fee. Distribution of hand-bills, bragging in the daily papers, and classing yourself as a Cheap John will perhaps bring more immediate results, but not a desirable or appreciative patronage, and will in the end be less profitable than a practice conducted on an honorable, a dignified, and professional basis. We should remember that for steady illumination a lamp is better than a rocket, and that it is a lamentable fact that low-priced dentistry is synonymous with cheap and shoddy work. Unfortunately, it seems that this class has flourished and multiplied, and has also grown bolder, more injurious, and more unscrupulous. While the best and highest element of the profession has continued to advance, the lowest element has grown in numbers and descended to lower and more injurious methods. While the present law gives us a board of examiners, which in one sense gives us protection, yet in another sense I think it falls short of its purpose. The board of examiners fill their positions in a creditable way and according to law. I commend their ability but regret their restrictions. A man may pass the necessary examination, as the present law requires a certain knowledge of theory, etc. Here the power of our board ceases. No man is infallible, and so long as this is true, no body of men or examining board can seize or stop every unworthy man who seeks to practise our noble profession. As the law intends that our examining board should protect the public, they should have some authority to withdraw certificates from those who indulge in malpractice, as a license from the board not only gives a man the privilege to practise, but assures the public, in the sense of law, that he is worthy and competent. As the law stands, there is an element of the profession which needs the greatest possible amount of legal supervision, and against which the public stands in the greatest need of protection. There should be adequate legal restriction placed on the grossest malpractice, even though you have a certificate from the State board. I do not mean that you can or any one can enact a law that says any man shall not do cheap work or advertise in newspapers, or on *hand-bills,* but it can protect the public against flagrant malpractice, at any price, or even free of charge. It can say you shall not advertise false statements, nor can you advertise the same thing under different names and at different prices. It is a mistaken idea with a young man when he enters practice that he is forced to offer some unethical inducement to people to patronize him, and he fails to give value to himself, the profession or others, as he has power to give, when he announces to the public in *heavy type* that his prices are the very lowest and his work the very best to be had. With our largely increased numbers, which must of necessity greatly increase competition, I am sure much good and little harm would have resulted had it not been for the man who claims every virtue in his advertisement and performs but wreck and ruin in his work. Through this method a vast multitude of the human race are slaughtered by deception and incompetency. The fact that he is not in our class professionally, or that our patients seldom go to him, does not help the situation. His advertisements continually injure us all by educating the public mind down to a scale of prices to which no man can adhere, do good work, and make a living *all* at the same time. Such being the condition of a patient's mind, what courses are open to us? Very often, by explaining the facts and the difference between the two operations, we may be able to show our patient that our operation is worth every cent we propose charging, and that it is to their advantage not to patronize a man simply because he advertises the very lowest prices, as the idea of economy would be a gross deception. We may fail to convince them of this fact, and allow them to go in search of some dentist whose prices are more in accordance with those they have seen advertised in the newspapers, or, worst of all, we may weaken, and when the prospect of losing a patient presents itself, we may come down from our previously stated price with the request that, as this is a special price, they must not say anything about it. This is the worst method, because it directly tends to lower the standard of work of every one who resorts to such unprofessional methods. If we possess professional honesty we named as near as possible an estimate and fair price in the first instance, and to accept less means to be underpaid for the work. No man puts forth his best efforts or uses the best material for an operation when he feels that his compensation is not adequate.

There are two conditions relative to dentistry which have a tendency to encourage the quack and apparently make his way easy, while they have the opposite effect on the conscientious practitioner. They are the facts that slipshod and superficial work inflicts a minimum of pain on the patient, and that our work is slow to find us out. The individual does not realize the harm he is doing himself or the dishonor he visits upon the profession; the desire of accumulation has eaten into him and dwarfed his conscience until his life is not worth living, but is a curse to the profession. Habit becomes law to a very great extent, and bad habits prevent the action of beneficial laws. It is impossible for us to keep the organs of professional honesty and decision of character at work if we persistently ignore their promptings and turn our backs on their necessities. There is no just law that demands that a man should drive business away from himself, even though he be a dentist. Yet our guide has told us we have two reputations,—namely, one with the public and one with our professional brethren. As we often have to deal with patients of others, ethics forms a very prominent link in the chain of our professional duties. Very often a layman having grievances comes to consult you without giving the name of his regular dentist. Very often the grievance is in the imagination and not in the quality of work, and a few words prompted by professional honesty will send the patient back to his regular dentist in a much better frame of mind. You can often do this without exceeding the demands of fraternal duty. There are many instances where the patient of another brother comes under our observation and influence. Very often they come into our own office in company with our own patient, both ethics and justice with a small portion of common sense demand that we should make no remarks of insinuation or accusation against our professional brother, nor in any manner should we endeavor to win the patient by alienating him from his regular dentist. Sometimes another practitioner intrusts his patient to our care for some special service; we should be scrupulous not to undertake any work other than that specified. Our fellow-dentist has displayed his confidence in our integrity, and it would be a poor return to deliberately alienate the patient. It would be well to refrain as far as possible from criticising the work of others, as we do not always know the circumstances under which the work has been done, nor how much of the patient's tale is to be believed. Our first duty is to be true to ourselves, then we will be true to others, and be ready to always give our best service and at the same time be ready to give warning against incompetency, as the rule of ethics would be broken and we would forfeit all professional honesty if we should attempt to protect the quack or any one who should be guilty of malpractice. Let us all bear in mind the best way to advertise is to join your local and State Dental Society and take an active part. No man is sufficient unto himself; if he be a superior dentist he owes it to his profession to join the society and demonstrate his superiority; if he feels himself deficient, he should join the society, that he may make himself more proficient by exchanging ideas with his fellow-man. Honor and character always command admiration and secure respect. It is natural to believe in such men and have confidence in them.

The public can always better understand and appreciate a man's real character and ability by the manner in which he conducts himself towards his professional brother and competitor, and by his transaction of the seemingly common-place details of daily duty, than by his public exhibition of himself as a genius, a superior operator, or a "Cheap John." It seems to me that this lesson ought to teach a man to strive that he might possess professional honesty and decision of character that will make him a man that knows the commercial value of being honest and a gentleman, not a quack. Such decision will warrant success professionally, and means success as a man. Lacking in this respect means failure as a citizen, and will in the end mean defeat. That which we obtain through false pretence and negligence is always far less ours than that which we acquire by honest and diligent, persevering effort. It is not ease, but effort, not facility, but difficulty, that makes a deserving dentist. There is, perhaps, no station in life in which more difficulties have to be encountered and overcome before any decided measure of success can be achieved than dentistry. Those difficulties are, however, our best instructors, as our mistakes are often our best teachers. Wherever there is difficulty, the individual man must come out for better or worse. If a man be a man of determination, the difficulties will fall away of themselves. Often you hear a man say he would like to act differently and be more professional, but his competition is of a nature it will not permit it. The desire must ripen into purpose and effort; and one energetic attempt is worth a thousand aspirations. Facility and ease will come with practice, and strength and fortitude with repeated effort. Encounter with difficulties will end only when life ends.

To help yourself honestly, you must have hands to help others. Take into consideration the fact that the vast majority of those who make the dominant idea of the profession money getting always fall far short of affluence. Practice founded on professional honesty and decision of character and a sufficiently high conception of the true spirit of professional ethics, a true regard for the actual public welfare, and so large a measure of sound, practical common sense as to refrain from a "Blind Tiger" method of practising dentistry, will soon win for itself the approval of the intelligent portion of the community, and in due time will be generally respected and appreciated. But so long as the profession is unprotected from persons who are willing to deceive the ignorant and prevail on them to accept in return for money work which instead of proving a lasting benefit is but the product of unskilful hands hastily put together, the better element of the profession will remain at a disadvantage. The best men do and always will maintain the standard of an honest and scholarly profession, and it is unfair to them and every honest member of our profession to compel them to meet and overcome prejudices created by ignorance and fraud, which are practised by those who will allow the reflection of a silver dollar to blind the influence of conscience and honesty. Through the profession the certificate is given to practise, therefore we are supposed to see the danger first, and to demand that the profession be closed against those unworthy of the calling, and the public look to us for protection against these barnacles of ignorance and greed. If this were not a fact, our profession would have no legal recognition, and legal recognition makes this a fact. Lack of professional qualifications in the few engenders lack of confidence in all, and the public and profession both become losers. Honorable contest for public preferment makes all better by bringing into active operation the very best of our energies and skill. But this is true only when the competition is honest. There is a field for usefulness to humanity in our profession, the breadth and future possibility of which are not equalled by any other profession. And now, having assembled together for the purpose of the advancement of our profession, let us each, as we come in touch with our professional brother, strive from the first to do him good; for it is an undisputed fact, that as we help one another we advance the standard of our profession. Remember, we are paid in good coin when we adhere to ethical principles and unflinching honesty of action. Now, can you afford to do slipshod, evasive, hypocritical work? Can you afford to shrink, or make believe or practise pretence in any act of life? No, no; for all the time you are moulding yourself into a deformity and drifting away from professional honesty and what is right. What the world does and says about you are really no matter, but what you think and what you do are questions vital as fate.

In conclusion, I will say I have referred mainly to the mere pecuniary and worldly advantage of professional honesty and decision of character.

In closing, I will merely say I believe there is an advantage beyond this. I do not believe any achievement can be higher or any reward greater than that of the man who, after living a long life of professional activity, and when life's sun is almost set, can look back and see there nothing but honest work honestly done, high ideals of professional honesty and dignity of character lived up to, and, in spite of disappointments, failures, and mistakes, activities that resulted from the purest and most honest intentions and principles.

